# Carbon-based nanomaterials in biomedicine and technology

**DOI:** 10.3389/fchem.2026.1787224

**Published:** 2026-06-12

**Authors:** María F. Cuenca-Lozano, Aygun Mehdiyeva, Aynura Karimova, Mustafa Muradov, Lala Gahramanli, Sevinj Nuriyeva, Habiba Shirinova, Vugar Yagublu, Cristian Vacacela Gomez, Stefano Bellucci

**Affiliations:** 1 Departamento de Producción, Facultad de Ciencias Exactas y Naturales, Universidad Técnica Particular de Loja, Loja, Ecuador; 2 Department of Chemical Physics of the Nanomaterials, Baku State University, Baku, Azerbaijan; 3 Department Chemistry of High Molecular Weight Compounds, Baku, Azerbaijan; 4 Nanoresearch Laboratory, Baku State University, Baku, Azerbaijan; 5 Faculty of Physics, Chemical Physics of Nanomaterials, Baku State University, Baku, Azerbaijan; 6 Department of Surgery, Medical Faculty Mannheim, University of Heidelberg, Mannheim, Germany; 7 INFN-Laboratori Nazionali di Frascati, Rome, Italy; 8 Universidad Ecotec, Samborondón, Ecuador; 9 National Institute of Materials Physics, Bucharest-Magurele, Romania

**Keywords:** biomedical application, carbon nanotubes, carbon-based nanomaterials, fullerene, graphene

## Abstract

Carbon-based nanomaterials (CBNs), including carbon nanotubes (CNTs), fullerenes, and graphene (GN), have attracted significant attention due to their unique structural, electrical, mechanical, and biocompatible properties. Their high surface area, excellent biocompatibility, and tunable physicochemical characteristics enable a wide range of applications, particularly in biomedicine, electronics, energy storage, and optoelectronics. CNTs, with their tensile strength and electrical conductivity, have been extensively studied for tissue engineering, drug delivery, and supercapacitor electrode applications. Despite concerns over their biocompatibility and toxicity, surface functionalization has shown promise in improving their performance and safety. Similarly, fullerenes, owing to their closed-cage structures and high electron affinity, exhibit potent antioxidant and photodynamic therapy capabilities, making them suitable for cancer treatment and optoelectronic devices. GN’s high surface area and charge mobility make it ideal for energy storage systems such as supercapacitors and lithium-ion batteries, although its agglomeration tendency limits its practical use unless modified. This work provides a narrative synthesis of recent advancements and practical limitations, outlining future directions for biomedical and technological applications.

## 1 Introduction

CBNs have transformed nanotechnology owing to their structural diversity and distinctive physicochemical properties. Composed entirely of carbon, they exhibit high stability (Bayatsarmadi et al., 2017; Khabashesku, 2026), outstanding electrical (Xiang et al., 2025) and thermal conductivity (Jóźwiak et al., 2020; Xie and Wang, 2023), excellent mechanical performance (stiffness, strength, toughness) (Mohanty et al., 2025), and generally favorable biocompatibility with low toxicity (Bianco et al., 2011). Their sp^2^ hybridization confers hydrophobic character (Choudhary et al., 2014). Properties are closely tied to structure and hybridization state (Gao et al., 2024). These materials—such as fullerenes, CNTs, GN, and graphene oxide (GO)—consist of carbon atoms arranged in 0D, 1D, and 2D architectures. These characteristics enable advances across biomedicine, where representative uses include drug delivery (Liu et al., 2011), wound healing (Gaur et al., 2021; Kalashnikova et al., 2015), bioimaging (Atabaev, 2018; Gao et al., 2024), biosensing (Hwang et al., 2020), tissue engineering (Yücer et al., 2025), and anticancer therapy (Tang et al., 2022).

Beyond their growing role in biomedical research, CBNs have also gained significant industrial and economic interest due to their exceptional electrical, mechanical, and conductive properties. They have become economically significant for global industrial manufacturing, particularly in energy storage, electronics, and advanced composite sectors. Recent market analyses estimate the global CNTs market at approximately USD 3-4 billion in 2024, with projections reaching USD 8–9 billion by 2030, corresponding to compound annual growth rates of ∼14–15% (Grand View Research, 2024; Markets and Markets, 2024). It is supported by large-scale lithium-ion battery, electronics, and electric-vehicle manufacturing (Fortune Business Insights, 2024; Grand View Research, 2024). In parallel, the global GN market, estimated at ∼ USD 200 million in 2023, is projected to grow rapidly to ∼ USD 1.5–1.6 billion by 2030 (CAGR ∼35%), reflecting expanding industrial use in coatings, sensors, flexible electronics, and electrochemical energy systems (Grand View Research, 2023). Collectively, these global and regional market trends underscore the growing economic impact of CBNs and highlight their strategic importance in modern industrial manufacturing.

This review presents a narrative synthesis of CBNs (CNTs, fullerenes, GN, and derivatives) in biomedicine and technology. We conducted non-exhaustive searches in Scopus, Web of Science, and Google Scholar, using combinations of the terms CBNs, CNTs, fullerenes, GN, biomedical applications, optoelectronics, and energy storage. We included peer-reviewed articles in English or Spanish that described relevant properties, functions, or experimental/technological applications and excluded grey literature, patents, and documents without peer review. Study selection was based on title/abstract screening and full-text reading when necessary, prioritizing topical relevance and recency. Formal risk-of-bias tools were not applied because our aim was to integrate trends and key concepts rather than perform a quantitative evidence synthesis. Examples and case studies were chosen for their representativeness of the available evidence.

## 2 Carbon nanomaterials: structures, properties, and applications

### 2.1 Carbon nanotubes

#### 2.1.1 Structural and physicochemical features

CNTs are classified, based on their structure, into single-walled (SWCNTs) and multi-walled (MWCNTs) nanotubes. SWCNTs consist of a single GN sheet seamlessly rolled into a cylindrical structure, where the rolling direction is defined by the chiral vector (n, m). This vector determines both the nanotube diameter and its chiral angle, and plays a crucial role in governing the electronic properties. The diameter is given by:

$$\left.d=\left(a/\pi \right)\times \sqrt{(n^{2}}+\mathrm{nm}+m^{2}\right)$$

- where, a = 1.42×√3 Å is the lattice constant of the GN sheet.

Depending on the values of (n, m), SWCNTs are categorized as armchair (n = m), zigzag (m = 0), or chiral (n ≠ m). The electronic behavior of SWCNTs is directly linked to their chirality: when (n − m) is a multiple of 3, the nanotube exhibits metallic or quasi-metallic behavior; otherwise, it behaves as a semiconductor. This phenomenon arises from the electronic band structure of GN, in which the conduction and valence bands meet at the Dirac points. Upon rolling into a nanotube, periodic boundary conditions impose quantization of the allowed wave vectors along the circumferential direction. Metallic behavior occurs only when these quantized states intersect the Dirac points; otherwise, a band gap is introduced, leading to semiconducting characteristics.

In contrast, MWCNTs consist of multiple concentric GN cylinders and are generally described by two idealized structural models: the “Russian doll” model (nested concentric tubes) and the “parchment” model (a single GN sheet rolled multiple times). The multilayered architecture of MWCNTs provides enhanced mechanical robustness, chemical stability, and resistance to structural defects compared to SWCNTs (Anzar et al., 2020).

CNTs inherit the exceptional properties of GN, particularly their outstanding mechanical performance, which originates from the strong sp^2^-hybridized carbon-carbon bonding network. They exhibit extremely high tensile strength, stiffness, and specific strength, making them highly effective as reinforcing agents in composite materials. In biomedical engineering, these properties are particularly advantageous for enhancing the mechanical integrity, durability, and load-bearing capacity of biomaterials. As a result, CNTs have been widely explored in applications such as bone tissue engineering, scaffolds, orthopedic implants, prosthetic devices, and bone cements, where improved mechanical stability and long-term performance are critical.

#### 2.1.2 Yields of CNTs synthesis and functionalization: literature overview

**FIGURE 1:**
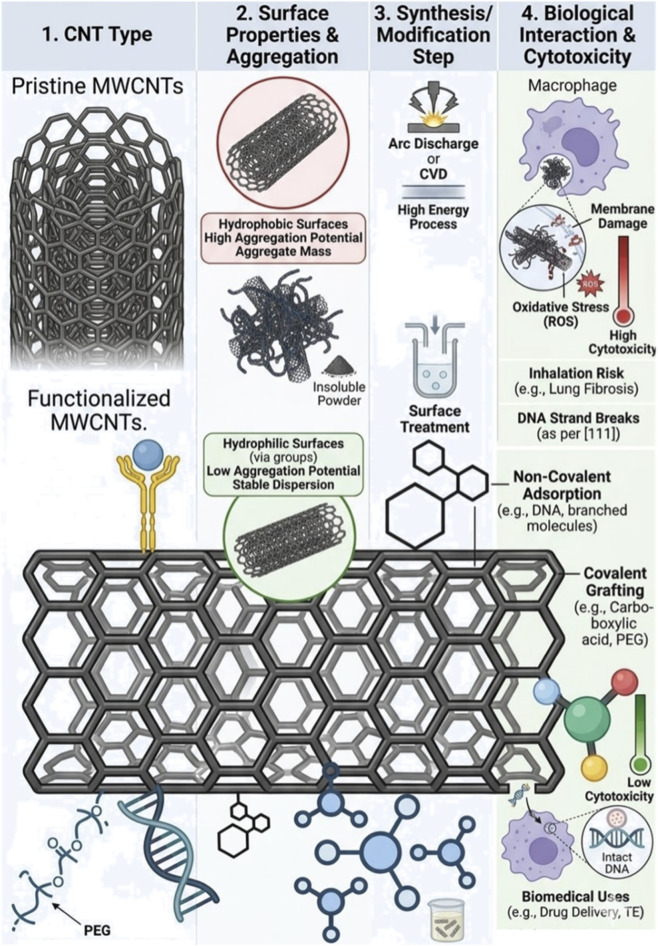
Schematic representation of functionalized CNT.

CNTs are commonly synthesized using arc discharge (Madikere Raghunatha Reddy et al., 2025; Shifa et al., 2024), laser ablation (Chrzanowska et al., 2015; Elahi and Zeinalipour-Yazdi, 2025), and chemical vapor deposition (CVD) (Unrau and Axelbaum, 2010; Yomogida et al., 2016), with CVD being the most widely adopted technique for scalable production. Post-synthesis functionalization or grafting is frequently employed to improve dispersibility and biocompatibility for biomedical applications (Speranza, 2021). The functionalization pathways of CNTs are presented in Figure 1. Pristine CNTs are inherently hydrophobic and tend to aggregate due to strong van der Waals and π-π interactions, resulting in poor colloidal stability in aqueous and physiological environments. Surface modification introduces hydrophilic functional groups (e.g., –COOH, –OH, –NH_2_) or polymeric coatings (e.g., polyethylene glycol, chitosan), which improve interactions with polar media and generate electrostatic and/or steric repulsion between nanotubes, thereby preventing aggregation and promoting stable dispersion. In addition, functionalization plays a critical role in modulating biological interactions. Surface coatings can reduce nonspecific protein adsorption, minimize direct membrane damage, and alter protein corona formation, leading to decreased cytotoxicity and improved cellular compatibility. Furthermore, functional groups provide active sites for the conjugation of drugs, targeting ligands, or biomolecules, enabling controlled delivery and enhanced therapeutic performance. Overall, surface functionalization not only stabilizes CNT suspensions but also tailors their physicochemical and biological identity for safe and effective biomedical use.

CNTs synthesis yields and functionalization efficiencies are reported in the literature using different quantitative metrics, including CNT fraction or purity in the as-produced material, carbon yield from the precursor, and mass productivity relative to catalyst loading. Similarly, functionalization efficiency is commonly expressed as degree of functionalization (DoF), concentration of surface functional groups (e.g., –COOH, mmol·g^−1^), or mass fraction of grafted organic layers determined by thermogravimetric analysis (TGA). Because these values depend strongly on synthesis route, catalyst system, reaction conditions, and analytical methodology, direct comparison across studies requires careful normalization. Table 1 summarizes representative yield ranges for major CNT synthesis methods and typical quantitative indicators of covalent functionalization, providing a concise reference for assessing scalability and surface modification efficiency.

**TABLE 1:** Representative literature ranges of CNT synthesis yields and functionalization efficiencies (2020–2025).

Process	Method	Performance metric	Key references
SWCNT synthesis	Laser ablation	CNT fraction in product ∼70–90%	Chrzanowska et al. (2015), Elahi and Zeinalipour-Yazdi (2025)
SWCNT synthesis	Arc discharge	SWCNT purity ∼30–55%; up to ∼80% in optimized fibers	Madikere Raghunatha Reddy et al. (2025), Shifa et al. (2024)
SWCNT synthesis	HiPco (CO–CVD)	Purity up to ∼97%; productivity ∼450 mg h^−1^	Unrau and Axelbaum (2010), Yomogida et al. (2016)
SWCNT synthesis	Fluidized-bed CVD	Carbon yield ∼28%	Li et al. (2021a)
MWCNT synthesis	Catalytic CVD	Productivity ∼20–50 g CNT gcat^−1^ h^−1^	Diao et al. (2018), Hussein et al. (2014)
Functionalization	Oxidative carboxylation	–COOH concentration ∼1–3 mmol g^−1^	Kolanowska et al. (2019), Pan et al. (2005)
Functionalization	Diazonium arylation	Degree of functionalization ∼0.2–1.0%	Schirowski et al. (2019), Yadav et al. (2022)
Polymer grafting	Click chemistry (PNIPAM)	Grafted polymer ∼15–34 wt%	Gohier et al. (2011)
PEG grafting	PEGylation	PEG content ∼10–20 wt%	Bottini et al. (2011), Siafaka et al. (2016)

* Widely accepted benchmark study retained for quantitative DoF calibration.

#### 2.1.3 Biomedical applications of CNTs

CNTs are also promising in tissue engineering (TE) due to tensile strength, electrical conductivity, large surface area, and biocompatibility features (Harrison and Atala, 2007; Rajakumari et al., 2022). However, they are not conventional biomaterials and require modification to address biocompatibility, toxicity, and biodegradability concerns. Surface functionalization (e.g., with chitosan, agarose, or collagen) reduces cytotoxicity and enhances tissue integration, producing flexible materials that interface with complex tissues, especially neural tissue; CNT-biopolymer composites are being explored for nerve guidance conduits at spinal lesion sites (Yim et al., 2025). Incorporating MWCNTs into polymeric scaffolds (e.g., collagen) enhances mechanical, physicochemical, and biological performance (Karbasi and Alizadeh, 2017; Tanideh et al., 2020; Zarei et al., 2020). For example, a biomimetic 3D scaffold (collagen/MWCNTs/curcumin) fabricated by freeze-drying method. The scaffold was designed to regenerate damaged tissues or organs by mimicking the structure and function of the native extracellular matrix (ECM). Such structure showed successful integration by FTIR/XRD, a porous interconnected architecture by SEM, and tensile strength improvement from 5 MPa to 19 MPa with 1% MWCNTs and 10% curcumin, alongside excellent wettability, bioactivity, and biocompatibility in SM-MSCs (*Skeletal Muscle-Mesenchymal Stem Cell*); *in vivo*, inflammation decreased after 6 weeks due to biodegradability and controlled drug release (Tanideh et al., 2020). Similarly, Farshidfar et al. (2022) reported that adding 0.5% (w/v) MWCNTs to P_3_HB scaffolds increased tensile strength from 2 MPa to 5 MPa and improved biocompatibility. Electrospun Gel/SWCNT/polyurethane nanofibers yielded vessel-like mechanical properties; SWCNTs increased Young’s modulus (16.47 ± 0.5 MPa) and ultimate tensile strength (23.73 ± 0.5 MPa), while gelatin increased elongation at break. Fiber diameter decreased from 210 nm to 140 nm, with improved early cell behavior; FTIR/DSC confirmed molecular interactions that enhanced hydrophilicity, and degradation was tuned by gelatin content. SEM and MTT showed dense myocardial myoblast and endothelial layers after 7 days, supporting cardiovascular TE potential. CNT-based scaffolds can also mimic neuronal electrical properties and facilitate neuronal stimulation.

**FIGURE 2:**
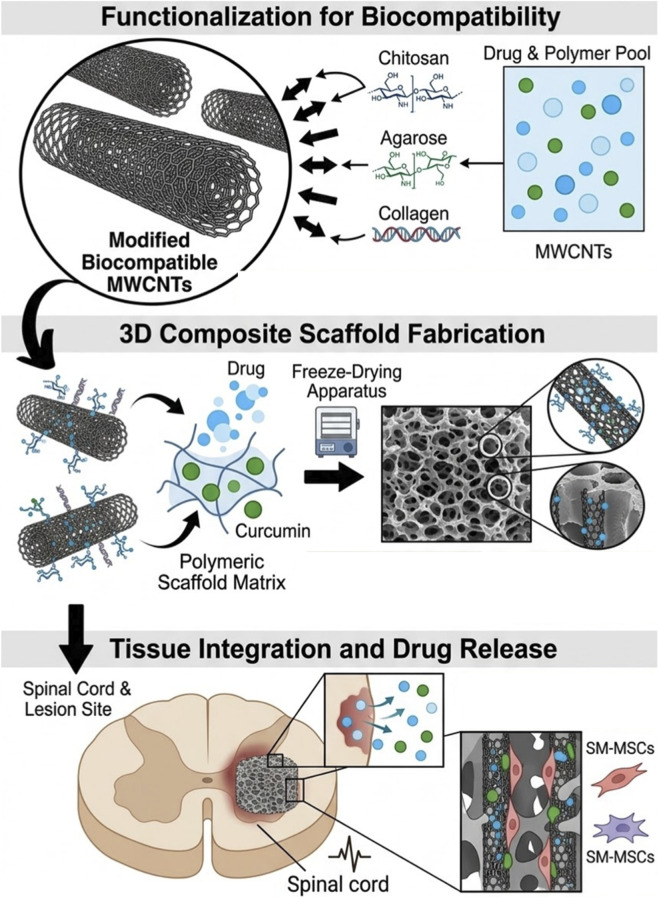
Schematic representation of the fabrication and biomedical application of functionalized CNT scaffolds.

CNTs offer high surface area-to-volume ratios for drug loading via π–π stacking, fluorescence detectability, and photoacoustic effects, enabling small carriers with high payloads and theranostic functionality (Wong et al., 2013). SWCNTs exhibit high specific surface area for loading proteins, DNA, and aromatic drugs via π–π and hydrophobic interactions (Battigelli et al., 2013). Drugs can also be covalently attached through ester or disulfide bonds for stimuli-responsive release and improved tumor targeting. Figure 2 schematizes drug loading in CNTs. Liu et al. (2007) showed PEG-functionalized SWCNTs loaded with doxorubicin (DOX) reduced toxicity and achieved significant antitumor effects after intravenous administration. Dai’s group (Wang et al., 2019a) reported DOX loading up to 4 g per g SWCNT via hydrophobic interactions and π–π stacking, with pH-responsive release and ligand-mediated targeting. To improve biocompatibility and biodegradability, Dahri et al. (Dahri et al., 2021) grafted DMAA-TMC onto functionalized SWCNTs, enabling controlled and targeted delivery. MWCNTs have also shown efficient drug delivery; BRC-conjugated MWCNTs induced apoptosis and necrosis in A549 and QU-DB lung cancer cells with no cytotoxicity toward MRC-5 cells, increasing Bax and decreasing Bcl-2 and achieving efficacy at lower doses than free BRC (Kamazani et al., 2021). Ultra-short CNTs (US-CNTs) can evade rapid RES clearance; cisplatin-loaded US-tubes exhibited greater cytotoxicity against MCF-7 and MDA-MB-231 cells within 24 h than free cisplatin (Guven et al., 2012).

Recent studies have explored hybrid nanostructures combining CNTs) with magnetic nanoparticles (MNPs), representing an emerging strategy for multifunctional nanomaterials. In particular, biologically sourced MNPs have attracted increasing interest for biomedical applications due to their intrinsic magnetic responsiveness, high surface area, and improved biocompatibility compared with many purely inorganic counterparts (Hong et al., 2024). Encapsulation of MNPs within the hollow interior of CNTs provides an effective approach to generate magnetically responsive nanostructures while preserving the external surface chemistry of the nanotubes. Such architectures can enhance nanoparticle stability by protecting the magnetic core from oxidation and degradation and by reducing direct exposure of metal surfaces to the biological environment, while maintaining strong magnetization suitable for magnetic targeting, imaging, or hyperthermia-based therapies (Joshi et al., 2024; Mishra and Yadav, 2024). For example, recent work has demonstrated an *in situ* one-step chemical vapor deposition method enabling the encapsulation of Fe_3_O_4_ and iron carbide nanoparticles (∼10–20 nm) within CNTs while preserving their room-temperature magnetic properties (Pizha et al., 2024). These magnetic CNT hybrids illustrate the growing potential of combining carbon nanostructures with magnetic cores to develop multifunctional nanocarriers and alternative conjugation strategies for advanced biomedical applications.

#### 2.1.4 Electronic, energy, and technological applications of CNTs

CNTs possess exceptional thermal conductivity due to efficient phonon transport along the tube axis in a defect-poor lattice, with anisotropy favoring axial over radial conduction; they can withstand ∼750 °C in air and up to ∼2,800 °C in vacuum. Thermal management is critical in electronics (CPUs, GPUs, smartphones, laptops) (Kalaiselvam et al., 2025; Marconnet et al., 2013; Nguyen et al., 2016), where operational stability typically requires 40 °C–90 °C. Polymers are attractive for electronics (low weight, flexibility, processability) (Abarrategi et al., 2008; Lee and Kim, 2025) but have low thermal conductivity and diffusivity; materials should have melting, glass-transition, and degradation temperatures above this range. Achieving >4 W m^−1^ K^−1^ in polymer composites without sacrificing processability remains challenging (Tanideh et al., 2020). Nanoscale fillers improve thermal, electrical, and mechanical properties (Farshidfar et al., 2022; Wong et al., 2013; Zarei et al., 2020); CNTs are prominent due to aspect ratio, strength, and high thermal conductivity (Zhang et al., 2015; Zhang H. et al., 2021). Percolated CNT networks in insulating matrices enhance electrical and thermal transport (Liu et al., 2007). Intrinsically, SWCNTs and MWCNTs reach ∼2000 and ∼3000 W m^−1^ K^−1^, respectively; MWCNTs are often preferred for thermal performance in polymer composites (Abarrategi et al., 2008). In PE-based nanocomposites plasticized with PEG, adding 15 wt% CNTs improved thermal conductivity by 68% versus neat PE and 100% over PE@PEG, with a transition from matrix-dominant to CNT-network-dominant heat conduction as loading increased; PEG improved processability and dispersion, while a uniform CNT network (TGA/SEM) enhanced interfacial phonon conduction (Konstantopoulos et al., 2021). Hybrid systems (e.g., PVDF with CNTs and GO) further improved dispersion and formed dense CNT/GO networks, reaching ∼0.95 W m^−1^ K^−1^ (Zhang et al., 2015).

Under mechanical stress, CNTs can bend, twist, kink, and buckle reversibly; defects limit this elasticity. SWCNTs can withstand pressures up to ∼24 GPa without significant defects; the elastic modulus is commonly analyzed (for MWCNTs also via TEM end-vibration methods) (Yaqoob et al., 2025; Ossila, 2026a). Owing to high carrier mobility, flexibility, and stability, CNTs are promising for flexible electronics (Wang et al., 2013a). Printing technologies enable large-area, low-cost devices compared with conventional microfabrication (Guven et al., 2012). Solution-processed SWCNTs have been used in printed circuits and devices (Cao et al., 2014; Xu W. et al., 2014; Yeom et al., 2015). SWCNT networks accommodate large strains via lateral deformation of entangled tubes (Lipomi et al., 2011; Meena et al., 2023) and have served as channel materials or electrodes in flexible/stretchable integrated circuits (Chandra et al., 2011; Chae et al., 2013; Xu F. et al., 2014), sensors (Cai et al., 2013; Cohen et al., 2012; Liang et al., 2013), OLEDs (Niu et al., 2013; Yu et al., 2009), supercapacitors (Feng et al., 2010; Rehman et al., 2022; Zhang et al., 2014), and touch panels (Pour et al., 2023). High conductivity (∼10^2^ S m^−1^), mechanical strength, and efficient ion/electron transport, together with unique structure–property relationships, favor diverse molecular electronic applications.

CNTs are key for next-generation energy storage owing to conductivity, strength, and efficient ion/electron transport, with applications in supercapacitors (Feng et al., 2010; Rehman et al., 2022; Zhang et al., 2014), batteries (Xiong et al., 2013), sensors (Ferrier et al., 2021; Rdest and Janas, 2021), wearables (Oh et al., 2022), and mechanical energy harvesters (Shah et al., 2009) as well as energy conversion (fuel cells, microbial fuel cells, solar cells). In supercapacitors, EDLC behavior emphasizes electrode surface area and stability (Wen et al., 2004). Pristine CNTs may have limited specific surface area and porosity, leading to lower wettability and ion access (Ding et al., 2018; Yang et al., 2019); surface inactivity and aggregation further hinder transport. Doping, activation, and irradiation can enhance performance. Boron doping modifies electronic structure and charge transfer (Wang et al., 2019b; Chen et al., 2024); sulfur doping increases interlayer spacing and ion accessibility (Chen et al., 2024). N-doped microporous carbon on CNTs achieved 310 F g^−1^ at 0.5 A g^−1^ and 18.6 Wh kg^−1^ with strong retention (Liu R. et al., 2020). KOH activation increased porosity and surface area, correlating with capacitance (Xu et al., 2008), with an optimal KOH:CNT ratio of 3:1 balancing high capacitance and rate capability (Ferry et al., 2022). Radiation-induced functionalization (Am-241, Sr-90, Co-60, Na-22) introduced oxygen groups; Am-241-treated CNTs (7.32% O) delivered 489.6 F g^−1^ at 0.1 A g^−1^, retained 98.5% after 5,000 cycles, and reached 56.9 Wh kg^−1^ at 9,992.2 W kg^−1^ (Kariper et al., 2022). A manganese sulfide-CNTs (MnS–CNTs) composite synthesized hydrothermally reached 1964.2 F g^−1^; a device with MnS, CNTs, and activated carbon achieved 240 C g^−1^, 53.3 Wh kg^−1^, and 7995 W kg^−1^ with stability over 1,000 cycles (Tamilselvan and Kundu, 2022). A comprehensive review covered CNT fiber fabrics, CNT/metal thin films, and remaining challenges for supercapacitors (Han et al., 2023).

Optical properties depend on chirality: semiconducting SWCNTs exhibit NIR photoluminescence with wavelength set by lattice orientation, while metallic CNTs and MWCNTs lack PL due to rapid nonradiative decay and inter-tube quenching, respectively. For transparent conductive electrodes (TCEs) in OPVs and OLEDs, ITO is common but brittle, costly, and inflexible (Han et al., 2023). CNT-based films (SWCNTs/MWCNTs) on glass or flexible plastics provide mechanical flexibility, high transparency, good conductivity, and roll-to-roll compatibility. Although sheet conductivity is lower than theoretical single-tube conductivity, performance comparable to ITO (<100 Ω/sq at >80% transparency) has been achieved, with superior flexibility and a work function (∼4.3 eV) favorable for hole injection/extraction. Advances in filtration, transfer printing, and densification yield smooth films compatible with ∼200 nm organic layers (Chien et al., 2010).

The Young’s modulus and tensile strength of SWCNTs and DWCNTs are approximately 1.2 TPa and 160 GPa, respectively (Ruoff et al., 2003; Tian et al., 2021), supporting electrode integrity under cycling in alkali-metal ion batteries. CNT paper can function as both active material and current collector in supercapacitors, reducing contact resistance and mass. Lithium storage in CNTs depends on morphology, defect density, and diameter; unlike graphite (LiC_6_ limit), CNTs provide more flexible Li^+^ accommodation due to tunable structures (Peng et al., 2008; Wang et al., 2025).

#### 2.1.5 Toxicity of CNTs

A major limitation of CNTs in biomedical applications is their limited biodegradability and potential for chronic toxicity (Luanpitpong et al., 2014). Pristine CNTs are highly hydrophobic and tend to persist in biological systems, which can reduce biocompatibility and promote long-term accumulation. Importantly, CNT toxicity is not an intrinsic property but depends strongly on their physicochemical characteristics, including size (diameter and length), aspect ratio, surface chemistry, degree of functionalization, purity, and aggregation state.

Among these parameters, diameter-dependent toxicity remains controversial. Some studies report that thicker and longer MWCNTs (e.g., 5–10 μm in length) induce greater DNA damage and lung inflammation, with increasing diameter (from 15 ± 5 to 30 ± 15 nm) correlating with higher toxicity (Fenoglio et al., 2012). In contrast, other reports suggest that thinner CNTs exhibit stronger cytotoxicity, possibly due to higher surface area and more efficient cellular uptake. For example, CNTs with diameters around 9.4 nm have been shown to induce more pronounced cytotoxicity and inflammatory responses compared to larger (∼70 nm) tubes, likely due to enhanced macrophage internalization. Conversely, some studies indicate that short MWCNTs with larger diameters (40–100 nm) may also exhibit elevated toxicity (Yuan et al., 2019). These discrepancies highlight that diameter alone does not determine toxicity but acts in combination with other structural and chemical factors (Costa et al., 2016).

Length and morphology also play a critical role. Longer CNTs (3–14 μm) may induce “fiber-like” pathogenic effects, including frustrated phagocytosis and chronic inflammation, similar to asbestos-like behavior. However, shorter CNTs can sometimes trigger stronger acute inflammatory responses, including increased cytokine production (e.g., TNF-α) in macrophages. Thus, parameters such as aspect ratio, rigidity, and structural morphology can independently modulate cytotoxic and immunological outcomes.

In addition, surface chemistry and purity are key determinants of toxicity. Residual metal catalysts (e.g., Fe, Ni, Co) from synthesis can promote the generation of reactive oxygen species (ROS), leading to oxidative stress, inflammation, and cellular damage. Structural defects and surface functional groups further influence protein adsorption, cellular uptake, and biological interactions. Diameter-dependent curvature also affects surface reactivity and interaction with biomolecules, thereby influencing toxicity.

Importantly, surface functionalization is an effective strategy to mitigate CNTs toxicity. Pristine CNTs readily aggregate due to strong van der Waals interactions, limiting their dispersibility and increasing localized toxicity (Jain et al., 2011). Covalent and non-covalent functionalization introduces hydrophilic groups or coatings, improving colloidal stability, reducing aggregation, and altering biological interactions. For example, acid-oxidized MWCNTs with increased carboxylation have been shown to induce less liver damage and oxidative stress compared to pristine CNTs, which is attributed to reduced length, improved hydrophilicity, and enhanced dispersibility (Jain et al., 2011). Therefore, rational surface modification plays a crucial role in improving the safety profile of CNTs for biomedical applications.

#### 2.1.6 Molecular structure and functionalization

Fullerenes are highly symmetric closed-cage carbon clusters (typical diameters ≈0.7 nm) composed of pentagons and hexagons arranged with icosahedral principles; families include C_60_, C_70_, C_80_ and C_90_ (Popov, 2016). In C_60_, 12 pentagons and 20 hexagons form a curved network with predominantly sp^2^ bonding and local sp^3^ character at pentagons, limiting full π-delocalization and favoring addition reactions. Electron delocalization is incomplete, so C_60_ is not “superaromatic”; double bonds avoid pentagons to reduce strain, giving electron-deficient alkene reactivity and high stability from geodesic structure and electronic distribution.

#### 2.1.7 Biomedical applications of fullerenes

**FIGURE 3:**
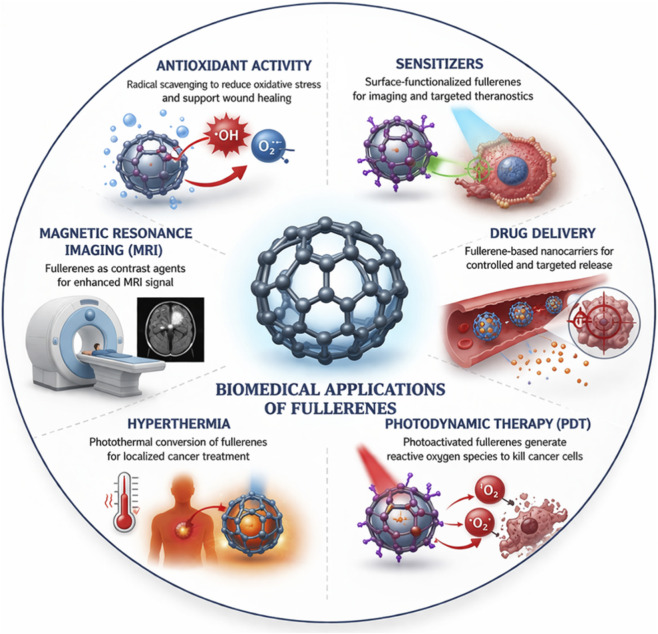
Biomedical application of fullerene.

Biomedical uses span imaging, drug delivery, hyperthermia, photodynamic therapy (PDT), and photoacoustic/thermoacoustic theranostics, with roles as MRI contrast agents, antioxidants, antibacterials, bone-targeting vectors and PDT sensitizers (Bakry et al., 2007; Chen et al., 2012; Fernandes et al., 2022; Ghiassi et al., 2014; Hamblin, 2018; Kawasaki et al., 2022; Liu et al., 2014; Lyon et al., 2006; Mirakyan and Wilson, 2002; Roy et al., 2018; Sun et al., 2016). The biomedical applications of fullerene are illustrated in Figure 3.

Beyond photovoltaics, potent radical scavenging underpins wound-healing acceleration; fullerenes often outperform CNTs as antioxidants by modulating ROS/RNS at injury sites (Cormas et al., 2026; Lee et al., 2013; Martinez et al., 2016; Shershakova et al., 2023). Antioxidant effects depend on molecular design: in HELFs, VI-419-P3K (C_70_-based) activated NRF2 and sustained protection, whereas GI-761 (C_60_-based) failed to activate NRF2 and upregulated NOX4, with cytotoxicity >20 μM after 72 h, highlighting dose/design sensitivity (Friedman et al., 1993; Liu et al., 2014). Fullerenols like C_60_(OH)_36_ scavenge lipid peroxyl radicals with pH-dependent activity and show synergism with PMHC; DFT points to radical addition on biphenyl-like regions as the dominant mechanism (Kostyuk et al., 2024). Interactions with RNase A indicate noncompetitive or mixed inhibition and reduced oxidative dityrosine/dimerization, supporting therapeutic potential (Tsai et al., 1997). Although pristine fullerenes are water-insoluble, both pristine and modified forms exert distinct bioeffects and should be evaluated separately; the well-known C_60_-in-olive-oil report showed lifespan extension in rats, albeit with small n (Aly et al., 2018).

Solubility/functionalization strategies (fullerenols, carboxyfullerenes, supramolecular complexation, albumin, PEG, sugars, cyclodextrins) improve dispersibility, biocompatibility and toxicity profiles (Radivoievych et al., 2023; Rašović, 2018). Examples include β-cyclodextrin conjugation for nuclear delivery and DOX–fullerene systems enabling controlled release at acidic pH (≈5.25) (Biswas et al., 2022). Hybrid platforms such as C_60_-IONP- PEG-FA for tumor targeting (biocompatible, ζ ≈ −27.3 mV; IONP 5–10 nm) and pluronic F127–CS nanoparticles (C_60_-PCNPs) enhance solubility, uptake, and combine PDT/microwave or thermal therapies, markedly reducing PC-3 survival versus controls (Lu et al., 2009; Shi et al., 2014).

#### 2.1.8 Electronic and technological applications of fullerenes

In electronics/optoelectronics, pristine fullerenes are insulating with low mobilities (≈10^−4^–10^–3^ cm^2^·V^−1^·s^−1^), but alkali-metal doping yields semiconducting/superconducting phases; strong electron affinity (≈2.6–2.8 eV; C_60_≈2.65–2.70 eV) and tunable LUMO levels make them efficient acceptors for OPVs, OFETs and photodetectors (E, 2017; Jebnouni et al., 2025). Polymer/fullerene nanocomposites enable LEDs, solar cells and sensors with low-cost processing; OLEDs benefit from charge-injection layers and energy-level matching (e.g., PEDOT:PSS), achieving high luminance and efficient recombination (Chen et al., 2022; Hamblin and Yoon, 2011; Hamid et al., 2021; Huang et al., 2014; Kang et al., 2012; Kausar et al., 2022). In OPVs, donor–acceptor blends such as P3HT/PCBM reach PCEs ≈3–4% with annealing-optimized morphology; PEDOT:PSS-based stacks report up to ≈9% under certain conditions (Bandaccari et al., 2018; Castro et al., 2018; Omrani et al., 2022; Redondo-Obispo et al., 2020; Vaagensmith et al., 2017). Functionalized fullerenes retain key acceptor properties with improved solution processability, supporting high-mobility OFETs; device performance depends on contacts, dielectric traps (surface–OH), and atmosphere, favoring inert-environment measurements or passivated dielectrics (Paukov et al., 2023; San et al., 2015; Wang et al., 2020). PCBM and related methanofullerenes commonly reach mobilities up to ≈0.2 cm^2^·V^−1^·s^−1^ after optimization (Wang et al., 2020).

Mechanically, C_60_ shows high effective bulk modulus upon compression (up to ≈668 GPa at 75% volume), shape recovery beyond 3,000 atm, and distinct intermolecular behavior (slick surfaces, relatively low melting points) (Thang and Kien, 2024). Thermal conductivity is low (C_60_ ≈ 0.2 W·m^−1^·K^−1^) compared with GN (≈5000 W·m^−1^·K^−1^); defects and low-frequency phonons limit heat transport (Ossila, 2026b; Ossila, 2026c).

PDT mechanism: upon UV/visible excitation, intersystem crossing to the triplet state enables ROS generation (^1^O_2_ in nonpolar media; O_2_^−^•/•OH in polar media with reductants like NADH). High triplet yields, photostability, and tunable derivatization make fullerenes versatile photosensitizers for cancer therapy (Freitas and Hamblin, 2016; Mroz et al., 2007). Drug-carrier roles leverage multivalent loading and controlled release; conjugation improves solubility and reduces systemic toxicity versus free drugs (Biswas et al., 2022; Kong and Zepp, 2012).

At a pH of 5.25, drug release can be fully triggered, making these conjugates suitable for targeted delivery to acidic tumor sites; the hydrophobicity challenge is mitigated by ethylene glycol spacers that improve the water solubility of drug–fullerene conjugates. Shi et al. (2014) developed a multifunctional nanosystem (C_60_- IONP-PEG-FA) by functionalizing C_60_ and iron-oxide nanoparticles (IONPs) with PEG and folic acid for enhanced tumor targeting. The platform supported cancer diagnosis, radiofrequency-assisted thermal therapy, PDT, and magnetic targeting, with no significant toxicity *in vitro* or *in vivo*; FT-IR/UV-Vis confirmed FA conjugation, TGA indicated ∼19% PEG grafting, DLS/TEM showed monodisperse 100–200 nm aggregates with ζ ≈ −27.3 mV, and TEM verified 5–10 nm IONP deposition on C_60_. Radivoievych et al. (2023) encapsulated fullerenes in pluronic F127–chitosan nanoparticles (C_60_-PCNPs), achieving high aqueous solubility, good uptake, and low toxicity; under 2.7 GHz microwave exposure against PC-3 cells, C_60_-PCNPs reduced survival to 7.5%, versus 42.2% with microwave alone, and to 32.5% with water-bath heating plus C_60_-PCNPs. Despite such advances, pristine fullerenes remain limited biologically due to poor water solubility. To address this, covalent modification and supramolecular complexation strategies improve dispersibility and biocompatibility, including dendritic amphiphiles (Hahn et al., 2012) and calixarene-based assemblies (Zhang TX. et al., 2021), albumin complexation (Cantelli et al., 2022) and PEGylation (Piotrowski et al., 2021), sugar-based polymer encapsulation (Murthy et al., 2002), ascorbic-acid adsorption (Santos et al., 2008), and fullerene–paclitaxel conjugates (Noori et al., 2025). These approaches afford controlled size, better compatibility, and optimized toxicity profiles, enabling biomedical use. Mesoporous silica nanoparticles coated with fluorescent C_60_-TEG-COOH yield water-soluble, biocompatible nanocarriers that support pH-sensitive release and fluorescent imaging; *in vitro* tests confirmed excellent biocompatibility (Tan et al., 2016).

### 2.2 Graphene

#### 2.2.1 Structural characteristics and surface chemistry

GN is a two-dimensional (2D) allotrope of carbon and the fundamental building block of graphitic forms. Unlike graphite, which consists of multiple GN layers stacked via van der Waals forces (Novoselov et al., 2005), GN is a single atomic layer of carbon atoms arranged in a hexagonal (honeycomb) lattice through sp^2^ hybridization. Each carbon atom has four valence electrons (1s^2^ 2s^2^ 2p^2^) (Gomes et al., 2022); three form in-plane σ bonds, and the fourth participates in π bonding, which underpins the material’s exceptional electrical conductivity. GN exhibits a zero band gap and ultrahigh charge-carrier mobility (up to 200,000 cm^2^·V^−1^·s^−1^) (Yusaf et al., 2022).

#### 2.2.2 Electronic, energy, and technological applications of GN

GN also offers a very high specific surface area (≈2,630 m^2^·g^−1^) (Gomes et al., 2022), making it an attractive electrode for energy-storage devices, particularly supercapacitors (Yusaf et al., 2022). However, pristine GN tends to agglomerate and restack, reducing accessible surface area and hindering ion transport, which impairs performance (Qiu et al., 2023; Wang Y. et al., 2021; Zhai et al., 2022). To address this, GN-based nanocomposites incorporating metal-oxide nanoparticles (NPs) have been developed to prevent agglomeration and enhance electrochemical behavior. Among these, RuO_2_ stands out for its conductivity and high theoretical capacitance. Integrating hydrous RuO_2_ NPs onto GN sheets mitigates restacking and introduces pseudocapacitance via redox reactions; for example, a RuO_2_/GN composite achieved an energy density of 20.1 Wh·kg^−1^ and retained 97.9% capacitance after 1,000 cycles, indicating stable, synergistic performance (Jang et al., 2003).

Beyond supercapacitors, GN-based nanocomposites are promising anodes for next-generation batteries. Wang et al. (2017) synthesized a GN/NiFe_2_O_4_/SnO_2_ ternary nanocomposite via a one-step hydrothermal route. As a Li-ion anode, it delivered a reversible capacity of 731.5 mAh·g^−1^ at 200 mA·g^−1^ after 50 cycles (80.9% retention), 613 mAh·g^−1^ at 800 mA·g^−1^, and 841.6 mAh·g^−1^ when the current density was reduced to 100 mA·g^−1^, reflecting excellent rate performance.

#### 2.2.3 Biomedical applications of GN-Based materials

GN-based composites also show strong potential in biosensing owing to high conductivity, surface area, and favorable biocompatibility. Zhuo et al. (2005) used a GN/Au NP hybrid to detect HBsAg with a 50 pg·mL^−1^ limit; BSA served as a negative control, confirming specificity. For small biomolecules, Popov et al. (2021) integrated reduced GN oxide (rGO) with polyaniline nanofibers for amperometric glucose sensing in human serum (limit of detection 0.089 mM). A GO/Au nanocluster-modified electrode enabled sensitive L-cysteine detection (LOD 0.02 μmol·L^−1^) (Vilouras et al., 2018). An ultrathin CS–GO platform on gold micro-gap electrodes allowed label-free, real-time monitoring of human dermal fibroblast proliferation—redox peaks at +300 and −300 mV increased from 1.923 to 11.195 nA over 96 h—and also supported glucose sensing from 1 μM to 20 mM (sensitivity 0.17 μA·mM^−1^).

Porous GN aerogels further enhance performance. Zhu et al. (2016) produced porous GN nanosheets (BET ≈3,100 m^2^·g^−1^) by chemically activating microwave-exfoliated graphite oxide, achieving a reversible capacity of ∼1,600 mAh·g^−1^ at 100 mA·g^−1^ due to a 3D network of interconnected meso/micropores. Ye et al. (2013) fabricated a 3D GN aerogel–nickel foam (GN@NF) electrode via freeze-drying and thermal annealing, delivering 366 F·g^−1^ at 2 A·g^−1^ with excellent cycling stability.

Electrons in GN exhibit long mean free paths, and its properties differ markedly from conventional metals/semiconductors. As a nonmetal, thermal energy is primarily transported by phonons (Liu H. et al., 2020). Phonons are scattered at defects, reducing thermal conductivity, yet they dominate specific heat across temperatures. Although GN’s specific heat has not been directly measured, estimates from graphite are used (Liu H. et al., 2020). The specific heat C = Cph + Cel; the phonon contribution increases with temperature (Liu H. et al., 2020; Ong and Pop, 2011). At room temperature, graphite shows Cph ≈0.7 J·g^−1^·K^−1^—∼30% higher than diamond—due to a higher density of low-frequency phonons from weak interlayer coupling; a similar trend is anticipated for monolayer GN, though substrates can modify values.

GN’s massless Dirac fermions yield phenomena such as the quantum Hall effect. Its thermal conductivity (∼5000 W·m^−1^·K^−1^) exceeds that of copper (Teng et al., 2017); grain-boundary scattering limits heat transfer, so conductivity scales with grain size and inversely with boundary energy. GN also exhibits a high Seebeck coefficient and thermoelectric figure of merit, supporting thermal-management and energy-harvesting applications (Hwang et al., 2022; Teixeira-Santos et al., 2023).

GN is highly amenable to chemical/biological functionalization, broadening biomedical applicability (Islam et al., 2024; Shen et al., 2012). Its intrinsic biocompatibility and scalable production make GN-based materials attractive for implantable devices (cardiovascular stents, orthopedic scaffolds, urinary implants) (Singh et al., 2024; Xue et al., 2022). SiO_2_ coatings enhance mesenchymal stem-cell adhesion, proliferation, and elongation on GN (Huo and Yue, 2020). Saravanan et al. (2018) reported a biodegradable CS scaffold with GO–Au nanosheets that improved cardiac conduction and contraction post-myocardial infarction in rats, increasing ejection fraction without immune activation.

GN nanosheets are readily internalized, enabling cellular delivery. With high conductivity, strength, and stiffness, GN and derivatives support tissue engineering for nervous, cardiac, and bone tissues (Bellet et al., 2021; Shin et al., 2016). For biomedical use, GO is often preferred over pristine GN because oxygenated groups (–OH, –COOH, epoxides) make it hydrophilic and stable in physiological media (Goenka et al., 2014), enabling drug/gene delivery (Goenka et al., 2014; Jiříčková et al., 2022), biosensing (Jiang, 2011), tissue engineering (Bellet et al., 2021; Shin et al., 2016), bioimaging (Loh et al., 2010), and wound healing (Shariati et al., 2023). Abundant functional groups allow conjugation of polymers, therapeutics, targeting ligands, and imaging agents; GO can load cargo via chemical coupling and physisorption, and functional groups enable attachment of DNA, proteins, quantum dots, and Fe_3_O_4_ NPs (Arkowski et al., 2020; Huang et al., 2025). Compared with pristine GN—which may induce inflammation or membrane disruption—GO often shows reduced cytotoxicity and supports π–π stacking, hydrogen bonding (Ghulam et al., 2022), and electrostatic interactions for controlled, stimuli-responsive release. Like other CBNs, GO can generate ROS after uptake (Jacinto et al., 2018) and exhibits immunomodulatory effects (e.g., macrophage activation, mitochondrial disruption) (Sims et al., 2017).

PEGylation improves GO biocompatibility and circulation. PEG–GO efficiently delivers aromatic, poorly soluble anticancer drugs (e.g., DOX) *in vitro* (Chitphet et al., 2019; Wang et al., 2019c); PEGylated nano-GO/DOX also enables combined chemotherapy–photothermal therapy with no cytotoxicity to EMT6 cells in safety studies. For osteosarcoma, polyhydroxy-glycerol–grafted GO carriers showed pH-sensitive delivery; electrochemically conjugated DOX exhibited strong antiproliferative activity with minimal *in-vivo* toxicity, aided by enhanced loading via polyglycerol branches (Ashrafizadeh et al., 2022; Targhazeh et al., 2023). A GO-incorporated CS/PVP electrospun scaffold improved 5-fluorouracil delivery, showing uniform morphology, good CaCo-2 biocompatibility, and higher cancer-cell kill versus GO-free controls (Grant et al., 2024). A multifunctional GO platform with mPEG-PLA [DOX@GO (mPP) (1/0.5)] achieved 161 nm size, 6.3% loading, 70% encapsulation efficiency, strong colloidal stability, and—under 808 nm irradiation—markedly enhanced efficacy against TNBC (G2/M arrest, mitochondrial dysfunction, ROS generation, apoptosis), suppressing tumors and lung metastasis *in vivo* (Zhu H. et al., 2023). Dual-drug systems include CS-functionalized GO co-delivering camptothecin and 3,3′-diindolylmethane with enhanced efficacy and reduced toxicity (MCF-7) (Deb et al., 2018), and GO–PEG–FA targeting folate-receptor-positive cells for camptothecin delivery (37.8% loading; sustained, pH-sensitive release; selective activity in MCF-7/HepG2 with minimal J774 toxicity) (Mauro et al., 2018). PEG-grafted GO (PEG-GO) enabled pH-responsive DOX release (≈40% at pH 5.5 in 24 h vs. 15% at pH 7.4 in 48 h) and, when conjugated with Rituxan (anti-CD20), provided targeted therapy and NIR imaging, boosting inhibition from 20% (non-targeted) to 80% (Wang D. et al., 2021). CS-functionalized magnetic graphene (CMG) nanoparticles supported simultaneous DOX delivery and GFP- plasmid transfection with pH-responsive release (pH 5.1 > pH 7.4), and served as MRI contrast agents (Wang et al., 2013b). GO-based hybrids integrating magnetic responsiveness and stimuli-sensitive release offer theranostic potential (Zhu J. et al., 2023), including SPION- decorated GO with modified DOX for combined therapy and high-resolution MRI (Wahajuddin and Arora, 2012), a SPION/PLGA/GO carrier for IUdR that crossed the BBB and, under a 0.5 T field, improved drug accumulation and survival in glioma-bearing rats (Zhi et al., 2021), Fe_3_O_4_- embedded rGO for chemo-magnetic hyperthermia (Alkhayal et al., 2021), and aptamer-functionalized magnetic GO for paclitaxel delivery (95.75% loading, pH-responsive release, low L- 929 toxicity, selective MCF-7 cytotoxicity) (Hussien et al., 2018). Magnetic, mesoporous GO composites enable targeted chemotherapy of poorly soluble drugs (e.g., CPT; ∼20% loading; pH-controlled release; HeLa cytotoxicity) (Gao et al., 2020), and β-cyclodextrin- functionalized magnetic GO co-delivers DOX/epirubicin with field-assisted targeting and enhanced MCF-7 cytotoxicity while maintaining low intrinsic toxicity (Wang et al., 2015).

#### 2.2.4 Functionalization strategies to enhance GO/rGO performance in electronics, biomedicine, and environmental applications

**FIGURE 4:**
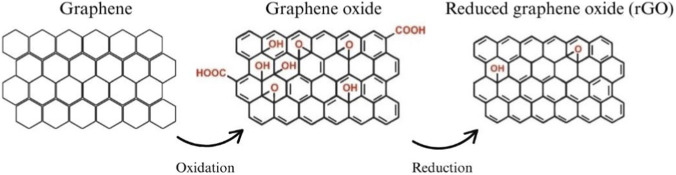
Structures of graphene (GN), graphene oxide (GO), and reduced graphene oxide (rGO).

Oxygenated groups disrupt the sp^2^ network, GO’s conductivity and mechanical integrity are reduced (Adotey et al., 2025). Chemical or thermal reduction to rGO partially restores the π system, improving properties (Abid et al., 2018). Thermal reduction above ∼900 °C is especially attractive because it avoids chemical reagents and enhances electronic characteristics. Figure 4 shows the structural differences between GN, GO, and rGO.

GO and rGO exhibit different optical bandgaps; the Tauc method is often used to estimate bandgaps from absorption vs. photon energy, guiding optimization for biosensors, optoelectronics, and energy storage (Abid et al., 2018). In biosensing, aryldiazonium-modified GN on glassy carbon with enzyme-induced Ag on Au NPs enabled sensitive BOT/E detection and is suitable for DNA platforms (Narayanan et al., 2015); GO-based electrochemical biosensors show strong promise for cancer diagnostics (Mohammadpour-Haratbar et al., 2023). In optoelectronics, NiO–GO thin films on flexible ITO/PET (spin-coated; GO via modified Hummers’ method) showed improved crystallinity, porosity, visible-range absorption, bandgap reduction from 3.88 to 3.50 eV, and better conductivity/resistivity with increasing GO content, indicating potential for flexible devices (Verma et al., 2025).

Dispersion and stability remain challenges: GO can aggregate in water, limiting surface area and recyclability; GN composites face hydrophobic-driven sheet aggregation and corrugation on substrates (Suter and Coveney, 2021). GO may induce dose-dependent cytotoxicity via ROS and oxidative stress (Gurunathan et al., 2019). To overcome these issues, GO is functionalized with polymers such as CS, PEG, PEI, PAA, and PVA, improving stability, biocompatibility, and dispersibility in aqueous/physiological media while protecting membranes from sharp-edge damage. CS improves mechanical strength and solubility of GO under acidic conditions; GO–CS shows pH-dependent particle size and ζ potential and, vs. PEGylated nano-GO, exhibits superior dispersibility in PBS and media with higher drug-loading capacity (Nayl et al., 2022). Adding GO to CS introduces extra–COOH groups for anionic sites, markedly enhancing adsorption of cationic dyes. GO@CS beads removed methylene blue with pseudo-second-order kinetics and Langmuir isotherms (q_max = 23.26 mg·g^−1^) and cleared 82.1% of dye from real wastewater in 135 min (Yadav et al., 2025). For protein delivery, CS- functionalized GO protected BSA and collagenase from proteolysis while maintaining activity; GO–CS shells limited enzymatic access, with proteins remaining intact after incubation at 37 °C, and gelatin zymography confirmed retained collagenase activity (Emadi et al., 2017).

For corrosion protection, a PVA/PANI/GO-COOH nanocomposite coating on cast iron (spin-coated) improved EIS impedance/capacitance and reduced Tafel corrosion rate in simulated seawater; oxygenated GO groups reinforced PVA, and PANI shielded pinholes/defects, yielding robust barrier properties (Elessawy et al., 2022; Ramezanzadeh et al., 2015). These results indicate PVA/PANI/GO-COOH as a durable protective coating for saline/marine environments.

## 3 Bioaccumulation of CBNs

An important consideration for the biomedical use of CBNs is their biodistribution and potential bioaccumulation following administration. After systemic exposure, these materials tend to accumulate primarily in organs associated with the reticuloendothelial system (RES), particularly the liver, spleen, and lungs. Their *in vivo* fate depends strongly on physicochemical characteristics such as particle size, surface functionalization, aggregation state, and administered dose. Particle size and hydrodynamic diameter are critical determinants of distribution and clearance. Smaller, well-dispersed nanomaterials may pass through biological barriers and, if below the renal filtration threshold, can be cleared via the kidneys. In contrast, larger or aggregated particles exhibit increased hydrodynamic size, promoting sequestration by macrophages and retention in MPS organs.

Surface modification strategies, including oxidation or polymer functionalization (e.g., PEGylation), can significantly influence circulation time, cellular uptake, and clearance pathways. In some cases, appropriately functionalized nanomaterials may undergo gradual elimination through renal or hepatobiliary routes, whereas poorly dispersible or highly aggregated particles may persist longer in tissues. Consequently, understanding biodistribution and potential long-term accumulation is essential for evaluating the safety profile of CBNs and for optimizing their design for biomedical applications.

Aggregation state and colloidal stability further modulate biodistribution. Aggregated nanomaterials exhibit reduced mobility, limited tissue penetration, and prolonged retention, whereas stable dispersions facilitate more uniform distribution and improved clearance.

In addition, shape and aspect ratio influence cellular processing. High-aspect-ratio materials such as CNTs may undergo incomplete phagocytosis, resulting in prolonged residence time and potential chronic accumulation.

Therefore, bioaccumulation is not solely dependent on exposure but arises from the interplay between physicochemical properties and biological responses, including protein corona formation, cellular uptake, and clearance mechanisms. Rational design strategies that optimize size, surface properties, and dispersibility are essential to minimize long-term retention and improve the safety profile of CBNs. Table 2 shows the bioaccumulation patterns of various CBNs in different tissues and organisms, highlighting factors that influence their distribution and retention.

**TABLE 2:** Summary of in vivo bioaccumulation behavior and elimination characteristics of representative carbon-based nanomaterials in biological systems.

Material class	Main organs of accumulation	Clearance pathways	Typical time scale of retention	Key determinants	Key references
CNTs (SWCNT/MWCNT)	Liver, spleen (MPS/RES); lung after inhalation or intratracheal delivery; lymph nodes	Hepatobiliary clearance; partial enzymatic degradation (peroxidases); slow macrophage-mediated clearance	Days–weeks; pulmonary retention up to ∼60 days in mice (formulation-dependent)	Length/aspect ratio, diameter, aggregation state, surface functionalization (PEGylation), dose	Hou et al. (2025), Al Faraj et al. (2011)
GO/rGO	Liver and spleen dominant; kidney involvement for small GO; lung at high doses	Renal clearance for small, well-dispersed GO; hepatobiliary for larger sheets	Hours–days (renal) to weeks (hepatic), depending on lateral size	Lateral size, thickness, oxidation degree, surface charge, dose	Chen et al. (2023), Mao et al. (2016)
Fullerenes (C60 and derivatives)	Liver, spleen, lung; distribution altered by functionalization	Hepatobiliary clearance; faster elimination for hydroxylated/carboxylated derivatives	Days–weeks; slower for pristine C60	Chemical derivatization, solubility, aggregation, administration route	Hou et al. (2024), Sayes et al. (2004)
Functionalized CBNs (PEGylated, biomolecule-coated)	Reduced RES uptake; more homogeneous distribution	Enhanced renal and hepatobiliary clearance	Shorter systemic residence vs. pristine forms	Surface chemistry, stealth coatings, hydrodynamic size	Hou et al. (2025), Zhuang (2025)

Overall, bioaccumulation of CBNs is strongly dependent on their physicochemical profile; small, well-dispersed, and appropriately functionalized nanomaterials are more likely to undergo efficient clearance, whereas aggregation, high aspect ratio, and poor surface modification increase the risk of prolonged retention and potential chronic effects.

## 4 Advantages and limitations of CBNs

CNTs, GN and fullerenes are three major CBNs with distinct structures and properties. This summary highlights their key advantages and limitations, typical high-impact applications, and representative performance metrics, based on recent literature. Understanding these differences can guide material choice for specific applications (e.g., flexible electronics vs. drug delivery vs. structural composites). Below is a concise comparative table. Each material’s strengths, weaknesses, and metrics are given with references. Table 3 compares the structural, electronic, and biological properties of CNTs, fullerenes and GN highlighting their similarities, differences, and potential application.

**TABLE 3:** Compares the structural, electronic, and biological properties of CNTs, fullerenes and GN highlighting their similarities, differences, and potential application.

Material	Key advantages	Key limitations	Typical high-impact applications	Performance metrics (typical ranges)	References
CNTs	- High aspect ratio and tensile strength - Excellent electrical (≈104–105 S/m) and thermal conductivity - Tunable semiconducting/metallic behavior for transistors	- Chirality and bandgap variability device inconsistency - Tendency to bundle (dispersion issues) - Difficult large-scale alignment	- Composite reinforcement - High-performance electrodes (batteries, supercaps) - Nanoelectronics (FETs, interconnects)	- Tensile strength ∼50–100 GPa -Young’s modulus ∼1 TPa - Electrical conductivity ∼104–105 S/m (metallic CNT) - Thermal conductivity ∼1,000–3000 W·m^−1^ K^−1^	Dresselhaus et al. (2001), Notarianni et al. (2016), Thiruvengadam et al. (2021), Zhao et al. (2022)
Fullerenes	- Zero-dimensional and highly symmetric (good for isotropic properties) - Effective electron acceptors (useful in photovoltaics) - Good solubility and processability (dispersible in organics)	- Limited electrical/thermal conductivity (insulators unless doped) - Lower mechanical strength (<10 GPa) - Rapid recombination in photovolatics unless paired	- Organic photovoltaics (as electron acceptor) - Drug delivery (biocompatible carriers) - Lubricants and catalysts	- Electron mobility (in polymers) ∼0.01–1 cm^2^/V·s - Energy gap ∼1.5–2.0 eV (molecular) - Thermal conductivity ≪1 W·m^−1^ K^−1^ (insulating)	Notarianni et al. (2016), Collavini and Delgado (2018), Zhao et al. (2022)
GN	- Extremely high in-plane strength (∼130 GPa) and stiffness (∼1 TPa) - Record electrical mobility (∼104–105 cm2/V·s) - High transparency and flexibility for coatings	- Zero bandgap (limits logic switches) - Pristine production yields defects; environmental degradation (oxidation) - Tend to restack/aggregate	- Flexible electronics (RF transistors, transparent conductors) - Composites (added strength, conductivity) - Sensors and membranes	-Carrier mobility ∼104–105 cm^2^V−1s^−1^ (suspended GN) -Sheet conductivity ∼106 S/m for CVD GN - Optical transmittance ∼97.7% for monolayer	Dresselhaus et al. (2001), Notarianni et al. (2016), Novoselov et al. (2012), Zhao et al. (2022)

Table Notes: Quantitative metrics are typical values from literature. Values can vary with material purity, preparation method, and experimental conditions. For example, CNT electrical conductivity ranges from ∼10^2^ to 10^5^ S/m depending on alignment and chirality. If precise data are not reported, ranges are given or described qualitatively. For electronics and photonics applications, graphene and semiconducting CNTs are often preferred for their high carrier mobility and tunability (graphene for flexible RF devices, CNTs for channel transistors). For structural composites, CNTs and GN both offer superb reinforcement; the choice depends on dispersion and processing (CNTs for high-aspect-ratio load-bearing, GN for planar coatings). For biomedical uses, fullerenes are attractive as drug carriers or in photodynamic therapy due to their biocompatibility and electron-accepting nature, whereas GN can serve in biosensors and scaffolds if properly functionalized. In general, one should balance electrical/thermal needs against structural and chemical requirements when selecting among these CBNs.

## 5 Discussion

**FIGURE 5:**
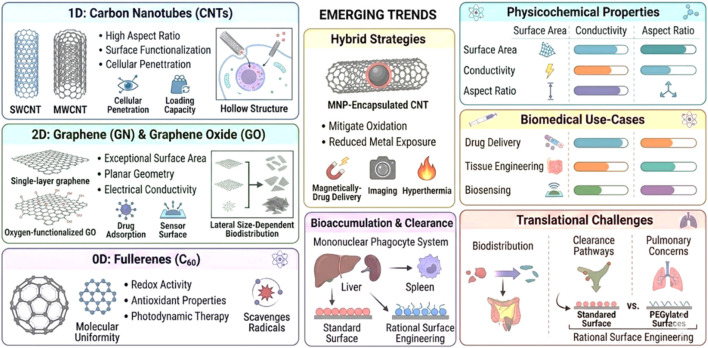
Comparative analysis of carbon-based nanomaterials (CBNs) for biomedical applications.

CBNs, including CNTs, GN and fullerenes, continue to attract significant attention due to their complementary physicochemical properties and broad applicability across biomedical and technological domains. Rather than a single “best” material, recent literature increasingly supports a context-dependent selection strategy, where performance is governed by morphology, surface chemistry, dimensionality, and interactions with biological systems (Fan et al., 2026; Hou et al., 2025; Zhuang, 2025). Figure 5 presents a comparative overview of CBNs used in biomedical applications.

CNTs remain particularly attractive for applications requiring high aspect ratio, mechanical reinforcement, and efficient cellular interaction, such as drug delivery, TE, and biosensing. Their hollow structure enables high loading capacity, while surface functionalization allows tuning of dispersibility, targeting ability, and biocompatibility (Alosime, 2023; Brito et al., 2024). Recent studies emphasize that the performance of CNTs is strongly governed by length, aggregation state, and degree of functionalization, with shorter, well-dispersed, and polymer-coated CNTs exhibiting improved biodistribution and reduced long-term retention (Babaei and Ganjalikhani, 2014; Bellucci, 2026). Compared with GN-based materials, CNTs often show superior penetration into cellular and tissue architectures; however, they also raise greater concerns related to pulmonary persistence following inhalation exposure, highlighting the importance of administration route and formulation design (Mohammad et al., 2025).

GN and GO represent a distinct class of two-dimensional CBNs with exceptional surface area, electrical conductivity, and chemical tunability. In biomedical contexts, GO offers advantages in drug adsorption, photothermal therapy, and biosensing, largely due to its oxygen-containing functional groups and planar geometry (Baruah et al., 2024; Li J. et al., 2021). However, comparative studies indicate that lateral size and thickness critically influence biodistribution and clearance, with small, well-exfoliated GO sheets showing partial renal elimination, while larger flakes accumulate predominantly in the liver and spleen (Chen et al., 2023; De et al., 2024). From a practical standpoint, GN-based materials often provide better scalability and batch-to-batch reproducibility compared with CNTs, supporting their use in large-area coatings, energy devices, and sensing platforms (Ozbey et al., 2025).

Fullerenes, although less structurally complex, exhibit unique molecular uniformity and redox activity, which underpin their antioxidant, photodynamic, and radical-scavenging properties (Gudkov et al., 2019; Mikheev et al., 2021). Functionalized fullerene derivatives have demonstrated promising biological compatibility and therapeutic potential; however, their relatively limited surface area and lower loading capacity compared with CNTs and GN constrain their use in applications requiring multifunctionality or high payload delivery (Lintaruk and Horbenyuk, 2025).

An emerging trend across recent studies is the development of hybrid and alternative conjugation strategies, particularly the integration of MNPs within carbon nanostructures. Encapsulation of magnetic cores inside CNTs represents a notable advance, as it mitigates nanoparticle oxidation, reduces direct metal exposure, and preserves magnetic functionality while leveraging the adsorption capacity and thermal conductivity of CNTs (Yan et al., 2019). Such magnetic CNTs exemplify how structural confinement and hybridization can overcome intrinsic limitations of individual materials and enable multifunctional platforms for magnetically guided drug delivery, imaging, and hyperthermia (Masotti and Caporali, 2013).

From a translational perspective, bioaccumulation and clearance remain central challenges for CBNs. Available *in vivo* evidence suggests that accumulation predominantly occurs in the mononuclear phagocyte system, with clearance pathways and retention times strongly influenced by size, surface chemistry, and aggregation state (Alexis et al., 2008; Babaei and Ganjalikhani, 2014; De et al., 2024). Importantly, recent studies indicate that rational surface engineering, such as PEGylation or biomolecular coating, can significantly reduce long-term retention and enhance clearance, reinforcing the necessity of controlled material design for clinical translation (Bellucci, 2026; Puiggalí, 2026).

Despite these advances, several key challenges continue to limit the large-scale industrial production and biomedical application of CBNs. In particular, achieving scalable and reproducible synthesis with precise control over structural parameters such as size, chirality, and defect density remains difficult, often leading to batch-to-batch variability and inconsistent performance. In addition, purification and quality control are critical issues, as residual metal catalysts, amorphous carbon, and structural defects can significantly affect both functionality and toxicity. The tendency of CBNs to aggregate during processing, storage, and in biological environments further complicates their stability and practical applicability. From a biomedical perspective, incomplete understanding of long-term toxicity, biodistribution, and clearance mechanisms continues to hinder clinical translation, while the lack of standardized characterization and toxicological evaluation protocols limits comparability across studies. Moreover, regulatory uncertainty and high production costs represent additional barriers to commercialization, particularly for applications requiring strict control over material composition, sterility, and biological interactions.

Overall, the comparative analysis presented in this review underscores that no single carbon nanomaterial universally outperforms others across all applications. Instead, optimal material selection must be guided by application-specific requirements, safety considerations, and scalability constraints. Future research should prioritize standardized characterization, long-term biodistribution studies, and hybrid material design, enabling informed decision-making and accelerating the responsible deployment of CBNs in biomedical and technological applications.

## 6 Conclusion

CBNs such as CNTs, GN, GO, fullerenes exhibit a wide range of extraordinary properties that arise from their distinct geometries and hybridization states. Their exceptional strength, conductivity, and surface characteristics open up avenues in numerous fields, including medicine, electronics, and energy.

However, challenges such agglomeration, biocompatibility, and scalable synthesis remain barriers to their widespread adoption. Continued research into functionalization, composite formation, and green synthesis approaches is essential to unlock their full potential. As the understanding of their structure-property-application relationships deepens, CBNs are expected to play a transformative role in future technologies. While this work synthesizes recent developments across biomedical and technological fronts, it does not apply formal risk-of-bias tools nor aim for exhaustive coverage. Future systematic assessments focused on specific subtopics, for example, *in vivo* toxicity of CNTs or the clinical effectiveness of fullerene-based photodynamic therapies would help quantify effects and enable rigorous comparisons.
